# Pattern of recurrence and prognostic factors in patients with *p*T1-3 N0 esophageal squamous cell carcinoma after surgery: analysis of a single center experience

**DOI:** 10.1186/s13019-019-0883-1

**Published:** 2019-03-12

**Authors:** Gang Lin, Haibo Liu, Jian Li

**Affiliations:** Department of Thoracic Surgery, Peking University First Affiliated Hospital, Peking University, Dahongluo Street 8, Xicheng District, Beijing, 100034 China

**Keywords:** Esophageal cancer, Prognostic factor, Survival analysis

## Abstract

**Background:**

The aims of this study were to determine the recurrence rate and the prognostic factors for recurrence-free survival (RFS) in esophageal squamous cell carcinoma (ESCC) patients without lymph node metastasis (LNM).

**Methods:**

Between January 2011 and June 2017, 101 patients with ESCC were treated and pathologically confirmed to be lymph node negative. The clinicopathological parameters were evaluated to identify the prognostic factors for RFS using Cox proportional hazards models.

**Results:**

Nineteen out of 101 patients (18.8%) developed recurrence, and the median RFS was 41 months. The most common pattern of relapse was local recurrence (*n* = 11; 57.9%), followed by distant recurrence (*n* = 7; 36.8%); one patient developed local and distant recurrence simultaneously. The results of multivariate analysis showed that the independent prognostic factors for decreased RFS in node-negative patients were a tumor located in the upper chest (odds ratio [OR], 0.767; 95% confidence intervals (CI), 1.523–14.916, *P* = 0.007), the presence of lymphovascular invasion (OR, 3.534; 95% CI, 1.077–11.596, *P* = 0.037), and a preoperative serum carcinoembryonic antigen level ≥ 5 μg/ml (OR = 5.466; 95% CI, 1.590–18.787, *P* = 0.007).

**Conclusions:**

The aforementioned parameters were the prognostic factors in node-negative ESCC patients, and they associated with a higher probability of recurrence after surgery. These patients should be followed closely, and adjuvant therapy should be considered.

## Background

Esophageal squamous cell carcinoma (ESCC) is a common malignant tumor occurring in Chinese individuals [1]. At present, esophagectomy plus systematic lymphadenectomy, with or without neoadjuvant chemoradiotherapy is the standard treatment for resectable ESCC [2]. Previous studies have reported several risk factors associated with the prognosis of esophageal cancer after radical resection [3–7]. Among these, lymph node metastasis (LNM) is considered as one of the most important determinants of the prognostic outcome, and the number of positive lymph node is regarded as one of the staging parameters in the American Joint Committee on Cancer (AJCC) Tumor Node Metastasis (TNM) staging classification for esophageal cancer [8]. Platinum-based chemotherapy has been reported to be beneficial in node-positive patients after surgery [9]. However, node-negative patients still have a high risk of recurrence after surgery, and it was reported that up to 40% of patients without LNM develop recurrent disease after surgery [10]. The prognostic outcome of these patients cannot be determined by the number of positive lymph node. Therefore, it is necessary to identify other clinicopathological parameters that could be used as prognostic indicators in these node-negative patients.

In the present study, we evaluated the recurrence rate and analyzed the prognostic factors for recurrence-free survival (RFS) in node-negative patients at a single institution. Our findings are likely to provide a reference point for clinicians to better assess the degree of tumor malignancy and to influence the use of adjuvant treatment in node-negative patients.

## Methods

### Patients

This study was approved by the Ethics Committee of the Peking University First Hospital. We retrospectively reviewed data from 301 patients with esophageal cancer (EC) who underwent esophagectomy for curative intent between January 2011 and June 2017 in the Thoracic Department. The following were excluded: (1) patients with a pathological type of nonsquamous cell carcinoma; (2) patients with pathologically confirmed LNM; (3) patients with tumors that had a positive margin (R1 or R2); (4) patients who received neoadjuvant or adjuvant chemotherapy or chemoradiotherapy; (5) patients who died of non-neoplastic causes; and (6) patients who were lost during follow-up.

### Preoperative staging procedures

Preoperative examinations, which included a thorough physical examination, a thoracic computed tomography (CT) scan, a bone scan, an endoscopic examination and a biopsy, a cervical ultrasound, an abdominal CT scan or an ultrasound, a cardiopulmonary function test, and a hematological profile, were performed to rule out distant metastasis and to evaluate the feasibility of surgery. Positron emission tomography–computed tomography (PET-CT) scans were not routinely performed in our department. They were only used for the preoperative examination of patients with highly suspected metastases, such as lymphadenopathy (> 1 cm in diameter on a CT scan).

### Surgical procedures

All operations were performed under general anesthesia and double lumen tracheal intubation by qualified surgeons. The surgery consisted of transthoracic esophagectomy and at least two field en-bloc lymphadenectomies (mediastinal and upper abdominal lymph nodes). Cervical lymph node dissections were only reserved for patients with suspected supraclavicular LNM before surgery. Gastric conduit was used for esophageal reconstruction in all patients, except one. The tumor was resected with a negative margin distance > 5 cm. For the resection of the upper ESCC, however, the distance of the proximal margin from the tumor might be < 5 cm, such that the proximal clearance was > 3 cm at least. There were two surgical approaches: (1) the right-sided transthoracic approach, where esophagectomy and mediastinal lymphadenectomy were performed through the right thoracic cavity. Stomach mobilization and abdominal lymph node dissection were performed at the abdominal stage. An anastomosis was made at the top right thoracic cavity or the left neck. The right-sided approach was used for cases of thoracic ESCC, regardless of the tumor location, except for those tumors located at the esophagogastric junction (EGJ). In the left-sided transthoracic approach, esophagectomy and thoracic and abdominal lymph node dissection were performed through the left thoracic cavity. An esophagogastric anastomosis was made underneath the aortic arch or at the left neck. The left-sided approach was used for cases of middle and lower ESCC and for tumors located at the EGJ. The extent of lymph node (LNs) dissection was as follows. During the left-sided procedure, the LNs at the paraoesophageal region (middle and lower paraoesophageal sites), the mediastinum (subcarinal, tracheobronchial, supradiaphragmatic, and posterior mediastinal sites), and the upper abdominal region (paracardial sites; LNs along the celiac, left gastric, and splenic arteries) were removed. During the right-sided procedure, in addition to the LNs that were dissected in the left-sided approach, the LNs along the bilateral recurrent nerves were also removed. After surgery, the specimens were examined by an experienced pathologist to determine whether LNM was present or absent and whether the surgical margin was free of the tumor (R0 resection). When the distance of the proximal margin from the tumor was less than 1 cm, the pathologist reported the precise distance between the surgical margin and the tumor.

### Collected clinicopathologic parameters

The following clinicopathologic parameters were collected:

1. General information: age, sex, smoking history, body mass index (BMI), preoperative serum carcinoembryonic antigen (S-CEA) level, and preoperative serum squamous cell carcinoma antigen (S-SCC) level.
2. Surgery-related information: surgical approach, operation time, blood loss during the surgery, and postoperative complications [11].
3. Postoperative pathology: tumor size, depth of tumor invasion (*p*T stage), total number of dissected lymph nodes (TLN), extent of lymphovascular invasion (VI), extent of perineural invasion (NI), tumor grade (well-differentiated (G1) /moderately differentiated (G2)/poorly differentiated (G3)), and TNM stage. The dissected lymph nodes were evaluated by well-trained pathologists. Each patient was assigned a pathological staging, according to the AJCC TNM Classification of Carcinoma of the Esophagus and Esophagogastric Junction (8th Edition) [8].

### Follow-up

Outpatient reviews or telephone follow-ups were conducted every 3–4 months for the first 2 years and every 6 months thereafter. The follow-ups were conducted until October 2018 or recurrence. Examinations included chest CT scans and abdominal and cervical ultrasounds. The collected information included the presence or absence of recurrence, date of recurrence, and site of recurrence. The diagnosis of recurrence was based on reports from radiographic tests. The overall survival (OS) was calculated from the operation to the last follow-up or death. The recurrence-free survival (RFS) was calculated from the date of surgery to the first recurrence. Based on the location of the relapse, recurrence was divided into locoregional recurrence or distant metastasis. Locoregional recurrence was defined as a recurrence restricted to the anastomotic site, the area of the original tumor bed or regional lymph nodes (including the cervical, mediastinum, and upper abdominal LNs). Distant recurrence was defined as any recurrence with features beyond those of a locoregional recurrence.

### Statistical analysis

SPSS 22.0 software (IBM Corporation, USA) was used for statistical analysis. Continuous variables were expressed as the mean ± standard deviation (SD) or median (range). Categorical variables were expressed as a percentage. All variables, including the demographic data, operative information and tumor characteristics, were analyzed. Kaplan–Meier methods were used to construct the RFS curves. The survival difference of each variable on RFS was analyzed by the log-rank test. The number of patients at risk was calculated for the beginning of each time period. Univariate and multivariate analyses were performed using the Cox proportional hazards regression model. Variables that had a significance level of *P* < 0.1 in univariate analysis were included in multivariate Cox regression analysis. All statistical tests were 2-sided, and a *P*-value < 0.05 was considered statistically significant.

## Results

### General characteristics of patients

There were 301 patients with EC who were treated with surgery during this period. The following were excluded: (1) patients with a pathological type of nonsquamous cell carcinoma (*n* = 8); (2) patients with pathologically confirmed LNM (*n* = 147); (3) patients with tumors that had a positive margin (R1 or R2) (n = 1); (4) patients who received neoadjuvant or adjuvant chemotherapy or chemoradiotherapy (*n* = 34); (5) patients who died of non-neoplastic diseases (*n* = 8); and (6) patients who were lost during follow-up (*n* = 2). As a result, 101 patients were enrolled in this study.

**Fig. 1:**
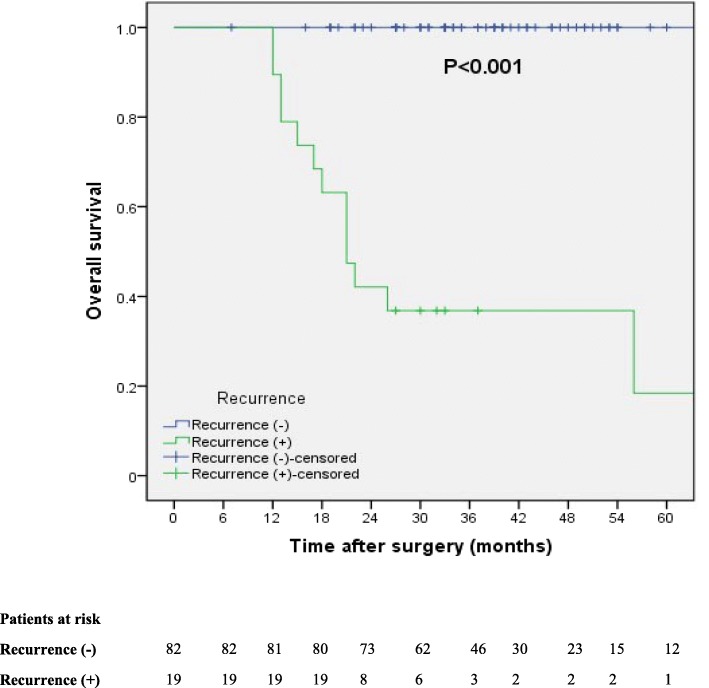
Overall survival curves for *p*N0 esophageal cancer patients stratified by recurrence (*n* = 101)

Of these patients, 78 were male (77.2%), with a median age of 64 years (range: 47–85 years). The mean S-CEA and S-SCC levels were 2.6 ± 1.5 μg/ml (range: 0.6–8.7 μg/L) and 2.5 ± 7.0 μg/ml (range: 0.3–70.0 μg/ml), respectively. The tumor was located in the upper third of the esophagus in 11 patients (10.4%), the middle segment of the esophagus in 58 patients (56.5%), and the lower third of the esophagus and the gastroesophageal junction in 32 patients (33.0%). Surgery was performed using left and right approaches in 44.3 and 55.7% of patients, respectively. Complications developed in 27.7% of patients after surgery. The TLN was 23 (8–56) for the entire study population. The median number of TLNs stratified by surgical approach was 23 (9–55) for the left-sided approach and 22.5 (8–56) for the right-sided approach, respectively. Among the 101 patients, 9 (8.9%) underwent cervical LN dissection or sampling, and the median number of removed LNs was 3 (1–9). All patients underwent thoracic and abdominal LN dissection, and the median numbers of removed LNs were 13 (1–41) and 9 (1–25), respectively. VI and NI were present in 5.9 and 14.9% of patients, respectively. The tumor grade was well differentiated (G1) in 16 patients (15.8%), moderately differentiated (G2) in 70 patients (69.3%), and poorly differentiated (G3) in 15 patients (14.9%). There were 51 patients (50.1%) with stage I disease and 50 patients (49.9%) with stage II disease. Among the 101 patients, 19 (18.8%) patients experienced recurrence. Compared with patients without recurrence, the prognosis of patients with recurrence was significantly worse (*P* < 0.001) (Fig. 1). The clinical characteristics of the patients are summarized in Table 1.

**Table 1:** Clinicopathological characteristics of node-negative patients (*n* = 101)

Characteristic	All (%)	Recurrence (+) (%)	Recurrence (−) (%)	*P*-value
	*n* = 101	*n* = 19	*n* = 82	
Age (year)^a^				
< 64	46 (45.5)	8 (42.1)	38 (46.3)	0.738
≥ 64	55 (54.5)	11 (57.9)	44 (53.7)	
Gender				
Male	78 (77.2)	18 (94.7)	60 (73.2)	0.065
Female	23 (22.8)	1 (5.3)	22 (26.8)	
Smoking history				
Present	50 (49.5)	10 (52.6)	40 (51.2)	0.762
Absent	51 (50.5)	9 (47.4)	42 (48.8)	
Surgical approach				
Left-sided	45 (44.6)	7 (36.8)	38 (46.3)	0.453
Right-sided	56 (55.4)	12 (63.2)	44 (53.7)	
Tumor location				
UT	11 (10.9)	5 (26.3)	6 (7.3)	0.041*
MT	58 (57.4)	11 (57.9)	47 (57.3)	
LT+ EGJ	32 (31.7)	3 (15.8)	29 (35.4)	
Tumor size (cm)^a^				
< 3	47 (47.5)	6 (31.6)	41 (51.2)	0.123
≥ 3	52 (52.5)	13 (68.4)	39 (48.8)	
*p*T Category				0.586
0	1 (1.0)	0 (0.0)	1 (1.2)	
1	30 (29.7)	4 (21.1)	26 (31.7)	
2	20 (19.8)	4 (21.1)	16 (19.5)	
3	50 (49.5)	11 (57.8)	39 (47.6)	
*p* TNM Stage				0.681
0	1 (1.0)	0 (0.0)	1 (1.2)	
IA_1_	8 (7.9)	2 (10.5)	6 (7.3)	
IA_2_	22 (21.8)	2 (10.5)	20 (24.4)	
IB	20 (19.8)	4 (21.1)	16 (19.5)	
IIB	50 (49.5)	11 (57.8)	39 (47.6)	

*EGJ* gastroesophageal junction, *LT* lower thoracic, *MT* middle thoracic, *TNM* the AJCC Tumor Node Metastasis Classification of Carcinoma of the Esophagus and Esophagogastric Junction (8th Edition); *UT* upper thoracic

^a^the median was used as the cutoff value

*:*P*-value < 0.05

### Recurrence patterns

Follow-up was performed until October 2018 or recurrence. The median follow-up time for the 101 patients was 41 months (range: 4–92 months). For the 19 patients who developed recurrence, recurrence (17 patients, 89.5%) usually occurred within the first three years after surgery. Recurrence peaked within the first year (8 patients, 42.1%) and then dropped rapidly after the third year after surgery (1 patient per year). The most common pattern of relapse was local recurrence (*n* = 11, 57.9%), followed by distant recurrence (*n* = 7, 36.8%); one patient developed local and distant recurrence simultaneously. The details of the recurrences are presented in Table 2.

**Table 2:** Distribution of recurrence sites

Recurrence site	No. of patients (%)
Locoregional recurrence	11 (57.9)
Mediastinal LN	5
Cervical LN	4
Anastomotic	2
Distant recurrence	7 (36.8)
Bone	3
Lung	2
Liver	1
Heart	1
Both	1 (5.3)
Mediastinal LN + liver	1
Total	19

*LN* lymph node

### Risk factors associated with recurrence in ESCC patients

The results of univariate analysis indicated that tumor location (*P* = 0.011), presence of VI (*P* < 0.001), CEA level (*P* = 0.003), and tumor grade (*P* = 0.011) were significantly associated with the RFS of patients. The results of multivariate analysis using the Cox proportional hazards regression model with stepwise selection also demonstrated that the independent prognostic factors for decreased RFS in patients with node-negative disease were a tumor located in the upper chest (*P* = 0.007, odds ratio [OR] = 4.767), CEA ≥5 μg/ml (*P* = 0.007, OR = 5.466) and the presence of VI (*P* = 0.037, OR = 3.534). The details are summarized in Tables 3 and 4.

**Table 3:** Univariate analysis of possible prognostic factors in node-negative patients (*n* = 101)

Possible prognostic factor	OR	95% CI	*P*-value
Age (< 64 yr. vs ≥ 64 yr)^b^	0.838	0.337–2.086	0.705
*p*-T stage (T_1_ vs T_2–3_)	0.569	0.188–1.721	0.318
*p*-TNM stage (I vs II)	0.642	0.258–1.602	0.342
TLN (< 23 vs ≥ 23)^b^	1.956	0.766–4.997	0.161
Sex (male vs female)	6.193	0.826–46.428	0.076
Tumor location (UT vs MT/LT + EGJ)	3.809	1.353–10.719	0.011*
Lymphovascular invasion (+ vs -)	7.471	2.428–22.990	< 0.001*
Perineural invasion (+ vs -)	2.365	0.848–6.591	0.100
CEA (≥ 5 μg/ml vs < 5 μg/ml)^a^	5.545	1.787–17.203	0.003*
Tumor grade (poor vs well + moderate)	3.577	1.335–9.581	0.011*
SCC (≥1.5 μg/ml vs < 1.5 μg/ml) ^a^	0.383	0.088–1.659	0.199
Surgical approach (left vs right)	0.581	0.226–1.499	0.262
Smoking history (yes vs no)	0.935	0.375–2.329	0.886
Postoperative complications (yes vs no)	1.145	0.435–3.015	0.784
BMI (< 23 vs ≥ 23)^b^	1.004	0.404–2.497	0.993
Operation time (< 445 min vs ≥ 445 min)^b^	0.951	0.377–2.397	0.915
Blood loss (< 200 ml vs ≥ 200 ml)^b^	0.493	0.143–1.699	0.262
Tumor size (< 3.0 cm vs ≥ 3.0 cm)^b^	0.445	0.169–1.173	0.101

*BMI* body mass index, *CI* confidence interval, *CEA* serum carcinoembryonic antigen level, *EGJ* gastroesophageal junction, *LT* lower thoracic, *MT* middle thoracic, *OR* odds ratio, *S-SCC* serum squamous cell carcinoma antigen level, *TNM* the AJCC Tumor Node Metastasis Classification of Carcinoma of the Esophagus and Esophagogastric Junction (8th Edition), *TLN* total number of removed lymph nodes, *UT* upper thoracic

^a^the upper limit of the clinical reference value was used as the cutoff value

^b^the median was used as the cutoff value

*: *P*-value < 0.05

**Table 4:** Multivariate analysis of independent prognostic factors in patients (*n* = 101)

	OR	95%CI	*P*-value
Tumor location			0.007*
UT	4.767	1.523–14.916	
MT/LT + EGJ	1		
Lymphovascular invasion			0.037*
Present	3.534	1.077–11,596	
Absent	1		
CEA (μg/ml)^a^			0.007*
≥ 5	5.466	1.590–18.787	
< 5	1		
Degree of tumor differentiation			0.132
Poor	2.289	0.780–6.717	
Well/moderate	1		

*CI* confidence interval, *CEA* serum carcinoembryonic antigen level, *EGJ* gastroesophageal junction, *LT* lower thoracic, *MT* middle thoracic, *OR* odds ratio, *UT* upper thoracic

^a^the upper limit of the clinical reference value was used as the cutoff value

*: *P*-value < 0.05

### Impact of different clinicopathological variables on RFS rate

**Fig. 2:**
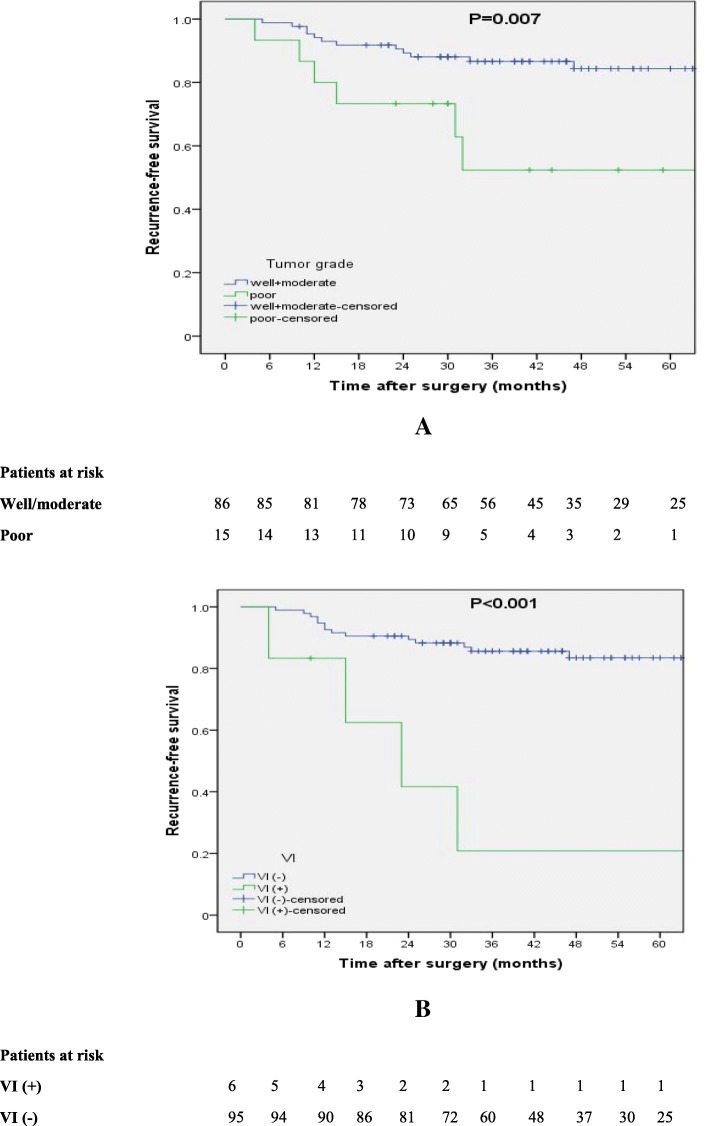
Recurrence-free survival curves for *p*N0 patients (n = 101) stratified by tumor grade (**a**) and lymphovascular invasion (**b**)

**Fig. 3:**
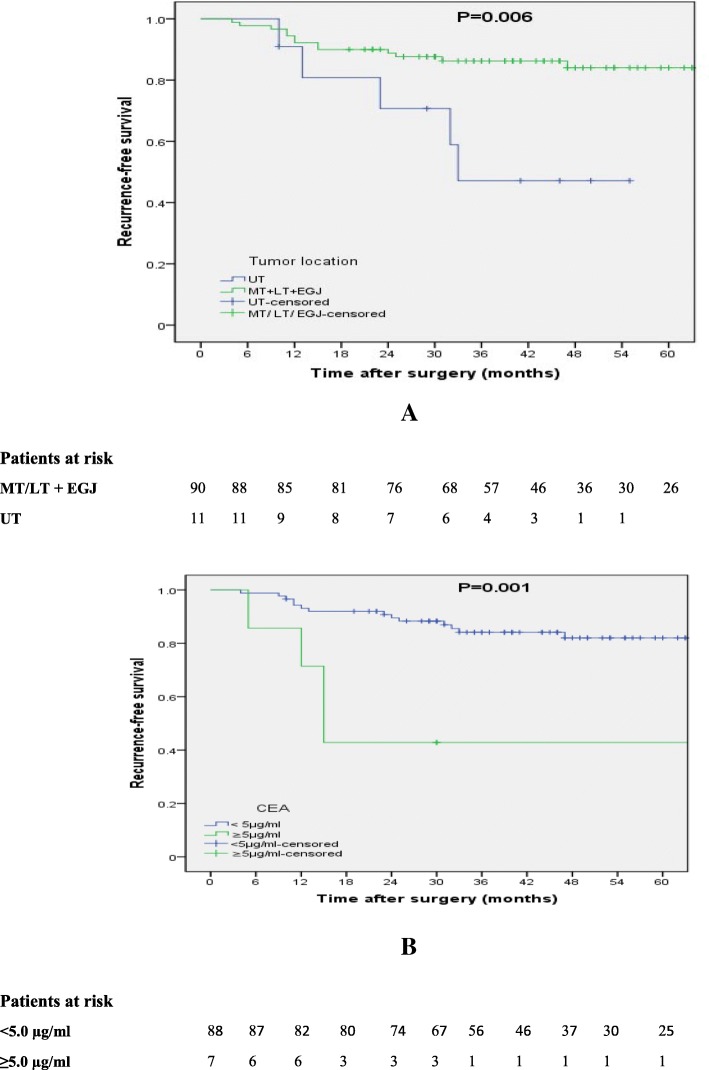
Recurrence-free survival curves for *p*N0 patients (n = 101) stratified by tumor location (**a**) and preoperative serum carcinoembryonic antigen level (**b**)

We estimated the 3- and 5- year RFS rates using the Kaplan–Meier method stratified by the aforementioned prognostic factors (Table 5). Poorly differentiated tumors had much worse prognosis than those with well- and moderately differentiated tumors in the log-rank test (*P* = 0.007) (Fig. 2a), although the degree of tumor differentiation was not an independent prognostic factor in multivariate analysis. In regard to the preoperative CEA level, a CEA level ≥ 5 μg/ml was strongly correlated with decreased RFS (*P* = 0.026) (Fig. 2b). Tumors located in the upper segment of the esophagus had a much worse prognosis than those located in the lower and middle chest (*P* = 0.006) (Fig. 3a). The presence of VI was also strongly correlated with the recurrence after surgery (*P* = 0.001) (Fig. 3b).

**Table 5:** Impact of clinicopathological parameters on RFS rates

Prognostic factor	No. of patients	3-yr RFS rate (%)	5-yr RFS rate (%)	*P*-value
Tumor location				
UT	11	47.1	0.0	0.006*
MT/LT + EGJ	90	86.2	84.0	
VI				
Present	6	20.8	20.8	< 0.001*
Absent	95	85.6	83.5	
Tumor grade				
Well/moderate	86	86.6	84.3	0.007*
Poor	15	52.4	52.4	
CEA (μg/ml)^a^				
< 5.0	91	84.1	82.0	0.001*
≥ 5.0	10	42.9	42.9	

*CEA* serum carcinoembryonic antigen level, *EGJ* gastroesophageal junction, *LT* lower thoracic, *MT* middle thoracic, *RFS* recurrence-free survival, *UT* upper thoracic, *VI* lymphovascularr invasion

^a^the upper limit of the clinical reference value was used as the cutoff value

*: *P*-value < 0.05

## Discussion

Recurrence was common for ESCC patients after esophagectomy. Previous studies have reported that the recurrence rate following curative radical resection by open thoracotomy ranged from 42 to 52% [12, 13]. The risk factors for recurrence included LNM, advanced stage, presence of VI, and location of the tumor [3–7]. Among these factors, LNM was considered the most important risk factor. It was reported that patients with LNM had a much higher recurrence rate than those without LNM [3]. However, even in ESCC patients without lymph node involvement, recurrence developed in several patients. In our study, the recurrence rate was 18.8% (19/101) in node-negative patients, which was consistent with the rates reported in previous articles [5, 10].

The present study evaluated the risk factors that influence the development of recurrence in node-negative patients after radical esophagectomy and showed that the presence of VI, a primary tumor located in the upper chest, and a higher S-CEA level were independently associated with decreased RFS after esophagectomy. The presence of VI and NI has been increasingly reported as an adverse prognostic marker in various malignancies [14–16]. A similar phenomenon was also observed by Huang and colleagues in ESCC patients [17]. The presence of VI and NI indicates that tumor cells have infiltrated into the lumina of lymphatic vessels and nerve sheath, which may lead to local spread and distant dissemination [14, 18]. Similar to these papers, we found that even in patients without LNM, the presence of VI suggested an increased probability of recurrence in ESCC patients after surgery.

In regard to tumor location, previous studies have reported conflicting results. Eloubeidi and colleagues [19] reported that tumors in the lower segment of the esophagus had a better prognosis. However, a large proportion of patients in his study had adenocarcinoma, and the results may not have reflected the exact impact of tumor location on prognosis. In another paper, Doki and colleagues [20] studied 501 patients with EC, most of which had ESCC. The authors reported that these patients had similar 5-year disease-free survival rate, regardless of tumor location. In our study, ESCC tumors located in the upper esophagus had a much worse prognosis than those located in the middle and lower chest, and the 5-year RFS rates were 0.0 and 84.0%, respectively (*P* = 0.006). We put forward two explanations for this: Firstly, almost all tumors could be completely resected, regardless of location, with the strict selection of surgical patients and the improvement of surgical techniques. However, for tumors located in the upper chest, it was more difficult to achieve a wide resection than for those located in the middle and lower chest. Secondly, several studies have reported that patients with ESCC tumors located in the upper esophagus have a higher rate of cervical LNM [21–23]. However, we routinely performed two-field lymph node dissection for thoracic ESSC patients, and cervical lymphadenectomy was reserved only for patients with suspected supraclavicular LNM before surgery for two reasons: (1) Cervical LN dissection associated with more postoperative complications, such as cord paralysis and aspiration [22, 23], and (2) the prognostic benefits of cervical lymphadenectomy remained inconclusive [22, 23]. The aforementioned reasons could explain the higher postoperative recurrence rate for patients with ESCC tumors located in the upper esophagus.

Tumor markers have been reported to associate with the prognosis of ESCC. Zhang and colleagues [24] studied 107 patients with locally advanced ESCC and found that cytokeratin-19 expression and the CEA levels were independent prognostic predictors for ESCC patients treated with concurrent chemoradiotherapy. Similarly, we found that an elevated S-CEA level associated with an increased recurrence rate. Kijima and colleagues [25] reported that stromal CEA expression plays an important role in the invasion of ESCC into the lymphatic system, which might explain the worse prognosis of patients with higher S-CEA levels. However, the S-SCC level was not recognized as an indicator for recurrence in our study.

Although the degree of tumor differentiation was not an independent prognostic factor in multivariate analysis, it was found to be a prognostic factor in the log-rank test (*P* = 0.007). The degree of tumor differentiation has been reported to be associated with the prognosis in ESCC patients. Situ and colleagues^5^ studied 317 ESCC patients with stage T2N0M0 and found that tumor grade was an independent prognostic factor (*P* = 0.011). In a study of 292 patients, it was reported that histologic differentiation was an independent prognostic factor for the survival of patients with ESCC (*P* < 0.001) [26]. The results we observed were similar to those described in the aforementioned papers. Several investigators have discovered that poorly differentiated tumors can synthesize additional cancer-promoting factors, which might explain the poor prognosis of patients with poorly differentiated esophageal cancers [27].

This study had several limitations. First, our study was a retrospective study with potential bias. Second, the number of patients included in our study was relatively small. Third, the follow-up time was relatively short, and long-term follow-up information was lacking.

## Conclusion

In conclusion, the presence of VI, a tumor located in the upper chest, and a preoperative S-CEA level ≥ 5 μg/ml were the prognostic factors in node-negative ESCC patients, and they associated with a higher probability of recurrence after surgery. These patients should be followed closely, and adjuvant therapy should be considered.

## Abbreviations

AJCC: American Joint Committee on Cancer
BMI: Body mass index
CEA: Carcinoembryonic antigen
CT: Computed tomography
EC: Esophageal cancer
EGJ: Gastroesophageal junction
ESCC: Esophageal squamous cell carcinoma
LN: Lymph node
LNM: Lymph node metastasis
LT: Lower thoracic
MT: Middle thoracic
NI: Perineural invasion
OR: Odds ratio
OS: Overall survival
RFS: Recurrence-free survival
SCC: Squamous cell carcinoma antigen
SD: Standard deviation
TLN: Total number of dissected lymph nodes
TNM: Tumor node metastasis
UT: Upper thoracic
VI: Lymphovascular invasion
